# Near Infrared Light Scattering Changes Following Acute Brain Injury

**DOI:** 10.1007/978-1-4939-3023-4_17

**Published:** 2015-06-22

**Authors:** David Highton, Ilias Tachtsidis, Alison Tucker, Clare Elwell, Martin Smith

**Affiliations:** 1 Neurocritical Care, University College Hospitals, Queen Square, London, UK; 2 Medical Physics and Bioengineering, University College London, Malet Place, London, UK; 3 Department of Medical Physics and Biomedical Engineering, University College London, London, UK; 4 NIHR University College London Hospitals Biomedical Research Centre, London, UK

**Keywords:** Near infrared spectroscopy, Brain injury, Scattering, Cerebral ischaemia

## Abstract

Acute brain injury (ABI) is associated with changes in near infrared light absorption reflecting haemodynamic and metabolic status via changes in cerebral oxygenation (haemoglobin oxygenation and cytochrome-c-oxidase oxidation). Light scattering has not been comprehensively investigated following ABI and may be an important confounding factor in the assessment of chromophore concentration changes, and/or a novel non-invasive optical marker of brain tissue morphology, cytostructure, hence metabolic status. The aim of this study is to characterize light scattering following adult ABI. Time resolved spectroscopy was performed as a component of multimodal neuromonitoring in critically ill brain injured patients. The scattering coefficient (*μ′*_*s*_), absorption coefficient and cerebral haemoglobin oxygen saturation (SO_2_) were derived by fitting the time resolved data. Cerebral infarction was subsequently defined on routine clinical imaging. In total, 21 patients with ABI were studied. Ten patients suffered a unilateral frontal infarction, and mean *μ′*_*s*_ was lower over infarcted compared to non-infarcted cortex (injured 6.9/cm, non-injured 8.2/cm p = 0.002). SO_2_ did not differ significantly between the two sides (injured 69.3 %, non-injured 69.0 % p = 0.7). Cerebral infarction is associated with changes in *μ′*_*s*_ which might be a novel marker of cerebral injury and will interfere with quantification of haemoglobin/cytochrome c oxidase concentration. Although further work combining optical and physiological analysis is required to elucidate the significance of these results, *μ′*_*s*_ may be uniquely placed as a non-invasive biomarker of cerebral energy failure as well as gross tissue changes.

## Introduction

The optical characteristics of cerebral tissues following acute brain brain injury (ABI) have been of considerable clinical interest, because they can be exploited to interrogate cerebral oxygenation in-vivo, non-invasively. An optical window in the near infrared spectrum (700–900 nm) facilitates measurement of the dominant absorbing chromophores in this region—oxy/deoxy-haemoglobin and cytochrome c oxidase, by their relative specific absorption spectra—thus inferring information about the haemodynamic and metabolic status of the brain [1]. However light transport through complex biological media is highly dependent on light scattering as well as absorption, which has not been comprehensively investigated following ABI, and may be an important confounding factor in the assessment of chromophore concentration changes [2], and/or a novel non-invasive optical marker of brain tissue morphology, cytostructure, hence metabolic status [3]. The aim of this study is to characterize light scattering in brain tissue following adult ABI.

## Methods

A total of 21 critically ill, ventilated brain injured patients were recruited following ethical approval and representative consent. Time resolved spectroscopy (TRS-20, Hamamatsu Photonics KK) was performed as a component of multimodal neuromonitoring in critically ill brain injured patients. Serial recordings were taken whilst in critical care. The reduced scattering scattering coefficient (*μ′*_*s*_), absorption coefficient, cerebral haemoglobin oxygen saturation (SO2) and total haemoglobin concentration ([HbT]) were derived by fitting the time resolved data (diffusion equation for light transport in a semi-infinite homogeneous medium, fitting the entire temporal point spread function, as standard within the Hamamatsu software). Three bilateral time-resolved recordings were made over frontal cortex at 4 cm source detector separation with a 5 s acquisition time. The mean of these three recordings were used for comparison. Cerebral infarction was subsequently defined on routine clinical imaging and paired comparison of *μ′*_*s*_ was performed in patients with unilateral infarction, using the paired *t*-test. *μ′*_*s*_

The wavelength dependence of *μ′*_*s*_ was analysed using least squares regression from the group data, across the three wavelengths measured (761, 801 and 834 nm), the 95 % confidence intervals and F-statistic is reported. To facilitate comparison with the literature three previously described models were fitted: $\mu {\hbox{'}}_s\left(\lambda \right)=a\lambda +b$, ${\mu}_s\left(\lambda \right) = b{\lambda}^{-a}$ normalized to the mean scattering value at 801 nm as in Matcher et al. [4], and $\mu {\hbox{'}}_s\left(\lambda \right)=b{\left(\lambda /500 nm\right)}^{-a}$ from Jacques et al. [5].

## Results

**Fig. 17.1:**
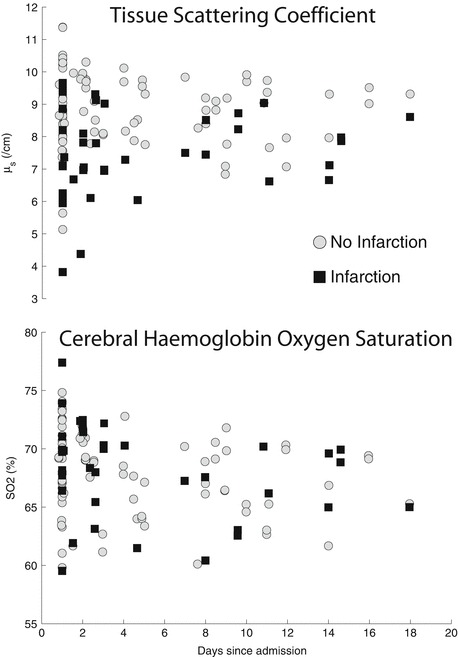
Mean *μ′*_*s*_ (834 nm) and SO2 for each day post intensive care admission in cerebral hemispheres demonstrating infarction versus no infarction. The lower scattering in infarcted cerebral hemisphere is evident, whereas there is no appreciable pattern with SO_2_

Patients’ characteristics are summarised in Table 17.1 and the *μ′*_*s*_ and SO2 data are summarised in Fig. 17.1. Ten patients suffered a unilateral frontal infarction, and mean *μ′*_*s*_ was lower over infarcted compared to non-infarcted cortex (Table 17.2). SO_2_ (injured 69.3 %, non-injured 69.0 % p = 0.7) and [HbT] (injured 64.6 μmol^−1^, non-injured 51.9 μmol^−1^ p = 0.09) did not differ significantly. The time course of *μ′*_*s*_ data is shown in Fig. 17.1 suggesting a trend of increasing *μ′*_*s*_ with respect to time in the infarct group:- however the patient group is heterogeneous, as is the onset of infarction.

**Table 17.1:** Patient characteristics. Reported as median [quartile] or number (percentage)

Age (years)	58 [47, 70]
Diagnosis	
Subarachnoid haemorrhage (%)	11 (52)
Intracerebral haemorrhage (%)	8 (38)
Ischaemic stroke (%)	1 (5)
Traumatic brain brain injury (%)	1 (5)
In-hospital mortality (%)	4 (19)

**Table 17.2:** *μ′*_*s*_ measured over infarcted and non-infarcted cortex

	*μ′*_*s*_761 nm (cm^−1^)	*μ′*_*s*_801 nm (cm^−1^)	*μ′*_*s*_834 nm (cm^−1^)
Infarct	7.43 (1.74)	7.18 (1.64)	6.87 (1.57)
No infarct	8.84 (1.51)	8.52 (1.45)	8.20 (1.43)
p-value	0.003	0.003	0.002

Brackets denote standard deviation

The wavelength dependence of *μ′*_*s*_ and fitted parameters are shown in Table 17.3. Using the linear model there was no significant difference in wavelength dependence defined by parameter *a* (injured −9.3 × 10^−3^, non-injured −9.2 × 10^−3^ p = 0.88)

**Table 17.3:** Wavelength dependence of *μ′*_*s*_ fitted to three established models

	$\mu {\hbox{'}}_s\left(\uplambda \right) = \mathrm{a}\uplambda + \mathrm{b}$	$\mu {\hbox{'}}_s\left(\uplambda \right) = {\mathrm{b}\uplambda}^{\hbox{-} \mathrm{a}}$	$\mu {\hbox{'}}_s\left(\uplambda \right) = \mathrm{b}{\left(\uplambda /500\mathrm{nm}\right)}^{\hbox{-} \mathrm{a}}$
a (cm^−1^ nm^−1^)	−0.0085 (−9.0 × 10^−3^ to −8.1 × 10^−3^)	0.77 (0.73–0.81)	0.77 (0.31–1.22)
b (cm^−1^ nm^−1^)	15.6 (15.3–16.0)	1485 (1134–1942)	12.4 (10.1–12.5)
F-statistic	1442	1424	11.14
p-value	<0.0001	<0.0001	0.001

Brackets denote the 95 % confidence limits of the model parameters. All models fit the data significantly

## Conclusions

Cerebral infarction is associated with significant reduction in *μ′*_*s*_ below previously reported values for normal adults [4]. Unilateral infarction was not associated with a similar difference in SO2 or [HbT] indicating either less discriminatory ability of these complex physiological variables or error in the assumptions underlying their derivation. The wavelength dependence of *μ′*_*s*_ was approximately linear over the narrow band of wavelengths studied and revealed similar parameter estimates to other reports of cerebral tissues in vivo [5] and in vitro [6]. This did not vary between injured and non-injured cortex. Clearly these findings have potentially important implications for analysis using differential spectroscopy over injured brain as variation in *μ′*_*s*_ violates assumptions required to calculate concentration changes.

**Fig. 17.2:**
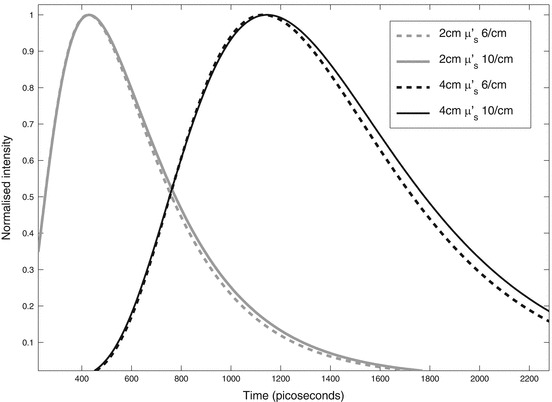
Two-layer model simulation of the effect of cerebral *μ′*_*s*_ on TPSF data at source detector separations of 2 and 4 cm. It can be seen that the TPSF has much greater sensitivity to *μ′*_*s*_ changes in the deep layer at 4 cm source detector separation than 2 cm. A two-layer semi-infinite slab geometry was used in this simulation, layer 1 (1 cm depth, *μ*_*a*_ 0.1/cm and *μ′*_*s*_ 10/cm), layer 2 (5 cm depth, *μ*_*a*_ 0.1/cm and *μ′*_*s*_ 6/cm or 10/cm) using a model from Liemert et al. [8]

The physiological origin of *μ′*_*s*_ reduction is also of considerable interest. Studies on exposed or in vitro cerebral cortex identify subcellular structures as the dominant scatterers in the near infrared spectrum with the mitochondria as a key contributor—given its typical size of 500–1000 nm. Thus scattering has been used experimentally to define mitochondrial volume and density—hence metabolic compromise [3]. Translation of this paradigm into the clinic might hold considerable promise, but it is important to consider the differences in measurement technique and underlying assumptions fitting the time resolved data. Specifically fitting the time resolved temporal point spread function (TPSF) assuming a homogenous semi-infinite medium may be an oversimplification of cerebral structure following brain brain injury and especially after surgical intervention. A specific issue which has been discussed is the sensitivity of the TPSF to *μ′*_*s*_ changes in the brain, rather than overlying extracerebral tissues. Simulated photon diffusion considering a two-layer structure and 2 cm source detector separation predicts little sensitivity in the TPSF to *μ′*_*s*_ below 1 cm depth [7] implying that much of our findings could be superficial tissue changes. However repeating simulations in a simple two-layer slab geometry shows greater sensitivity of the TPSF to changes in scattering to the 4 cm source detector separation we have used (Fig. 17.2).

Further work is required to identify the physiological significance of our findings. Individualized optical modeling based on anatomical data from radiological imaging is feasible, and may define the optical situation with further clarity. Combining optical and physiological modeling may hold the key to elucidate individualized physiological explanations for our multimodal optical and physiological data. Thus optical scattering might have considerable potential as a novel independent optical signal of cerebral ischaemia/infarction following ABI, by characterising the resultant changes in mitochondrial morphology and cellular structure in vivo—an approach which appears more discriminatory than traditional haemoglobin based measures.
